# Analysis of paracetamol, pseudoephedrine and cetirizine in Allercet Cold^®^ capsules using spectrophotometric techniques

**DOI:** 10.1186/s13065-018-0436-z

**Published:** 2018-06-01

**Authors:** Souha H. Youssef, Maha Abdel-Monem Hegazy, Dalia Mohamed, Amr Mohamed Badawey

**Affiliations:** 1 Pharmaceutical Analytical Chemistry Department, Faculty of Pharmacy, October University for Modern Sciences and Arts, 6 October City, 11787 Egypt; 2 Analytical Chemistry Department, Faculty of Pharmacy, Cairo University, Kasr El-Aini Street, Cairo, 11562 Egypt; 3 Analytical Chemistry Department, Faculty of Pharmacy, Helwan University, Ein Helwan, Cairo, 11795 Egypt; 4 Pharmaceutical Chemistry Department, Faculty of Pharmaceutical Sciences and Pharmaceutical Industries, Future University in Egypt (FUE), Cairo, 12311 Egypt

**Keywords:** Paracetamol, Pseudoephedrine, Cetirizine, Ratio subtraction–ratio difference, Successive derivative ratio, Derivative ratio spectra–zero crossing, Pure component contribution algorithm

## Abstract

Paracetamol (PAR), Pseudoephedrine hydrochloride (PSE) and cetirizine dihydrochloride (CET) is a ternary mixture that composes tablets which are popular for the relief of flu in Egypt. The spectra of the drugs were overlapped and no spectrophotometric methods were reported to resolve the mixture. This research proposes four spectrophotometric methods that are efficient and require water only as a solvent. The first method was ratio subtraction-ratio difference method (RSDM) where PAR was initially removed from the mixture by ratio subtraction and determined at 292.4 nm, then PSE and CET were quantified by subtracting the amplitudes of their ratio spectra between 257.0 and 230.0 nm for PSE and between 228.0 and 257.0 nm for CET. The second method was derivative ratio spectra—zero crossing (DRZC) which was based on determining both PSE and CET from the zero-crossing points of the first and third derivative of their ratio spectra at 252.0 and 237.0 nm, respectively while PAR was determined using its first derivative at 292.4 nm. Moreover, the ternary mixture was resolved using successive derivative ratio (SDR) method where PAR, PSE and CET were determined at 310.2, 257.0 and 242.4 nm, respectively. The fourth proposed method was pure component contribution algorithm (PCCA) which was applied to quantify the drugs at their λ_max_. Recovery percentages for RSDM were 100.7 ± 1.890, 99.69 ± 0.8400 and 99.38 ± 1.550; DRZC were 101.8 ± 0.8600, 99.04 ± 1.200 and 98.95 ± 1.300; SDR were 101.9 ± 1.060, 99.59 ± 1.010 and 100.2 ± 0.6300; PCCA were 101.6 ± 1.240, 99.10 ± 0.5400 and 100.4 ± 1.800 for PAR, PSE and BRM; respectively. The suggested methods were effectively applied to analyze laboratory prepared mixtures and their combined dosage form. 
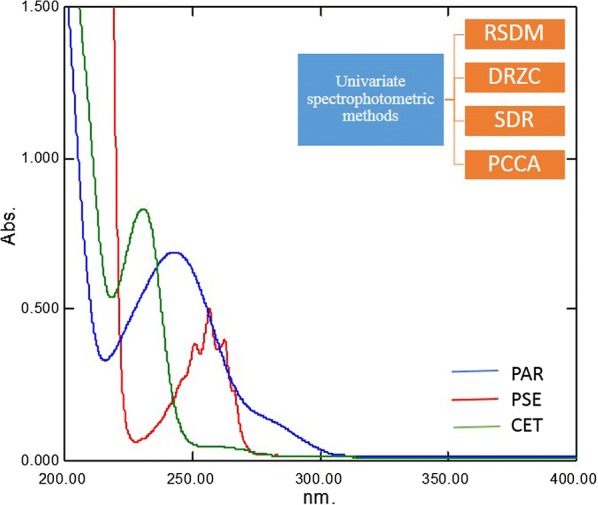

## Introduction

**Fig. 1:**
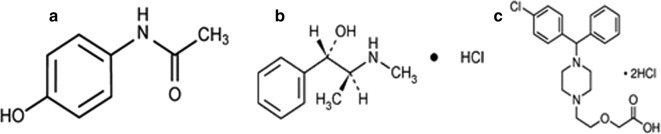
Chemical structure of **a** paracetamol, **b** pseudoephedrine HCl, **c** cetirizine 2HCl

The drugs under study in this research include paracetamol (PAR), pseudoephedrine HCl (PSE) and cetirizine dihydrochloride (CET). PAR (*N*-(4-hydroxyphenyl) acetamide) [1] is an analgesic and an antipyretic, used to treat many conditions such as muscle ache, tooth ache and arthritis [2]. PSE ((1*S*,2*S*)-2-(methylamino)-1-phenylpropan-1-ol hydrochloride) [1], is a nasal decongestant which acts by reducing inflamed membranes of mucosa, also it is used for bronchodilation [2]. CET ((*RS*)-2-[2-[4-[(4-chlorophenyl)phenylmethyl]piperazin-1-yl]ethoxy] acetic acid dihydrochloride) [1], is an antihistamine known for its stabilizing effect on mast-cells thus used in the treatment of allergies [2]. The ternary mixture is present in the Egyptian market as Allercet Cold^®^ and it is famous for its effectiveness in relieving symptoms associated with common cold, sinusitis and flu. The chemical structures of the three drugs are illustrated in Fig. 1.

Nowadays, effective cold treatments are on high demand especially for people with busy schedules and need to be alert and focused as fast as they can. This was successfully achieved by pharmaceutical companies by including more components in their formulations to treat more symptoms in one pill or capsule. Nevertheless, quality control lab analysts faced many challenges regarding the analysis of the more complex dosage forms, hence the development of novel analytical techniques was necessary. It was important to consider methods which were simple, rapid and low in cost without affecting accuracy and reliability of the results. The literature revealed many methods for the determination of each drug as a single component or in mixtures [3–9]. However, only two HPLC–UV [10, 11] methods for the determination of this combination were available. That being said, chromatographic methods consume time and solvents contributing in the high cost of method development and optimization which is disadvantageous for quality control laboratories. In addition, highly trained staff are required to operate the apparatus. On the other hand, mathematical spectrophotometric methods are considered faster and cheaper. Also, spectrophotometers are available in most labs and easier to operate therefore offering substitute resolutions for the complex mixtures of analytes without the need of prior separation or extraction [12]. The absence of any analytical approaches using spectrophotometry for the quantitation of this mixture has motivated us to develop spectrophotometric methods with good accuracy and precision for the analysis of the proposed combination. The methods utilized simple manipulation steps and did not require any sophisticated instruments using distilled water as a solvent which causes no environmental harm and safe for analysts in the field.

## Theoretical background

The methods applied for the analysis of the ternary mixture were ratio subtraction [13]—ratio difference [14] (RSDM), derivative ratio spectra–zero crossing [15] (DRZC), successive derivative ratio [16] (SDR) and pure component contribution algorithm [17] (PCCA). These methods are well developed and were successfully adopted for resolution of overlapped spectra of ternary mixtures.

## Experimental

### Apparatus and software

Shimadzu—UV 1800 double beam UV–Visible spectrophotometer (Japan) and quartz cells (1 cm) at a range of 200.0–400.0 nm was used for measuring the absorbance. Spectral manipulations were carried out by Shimadzu UV-Probe 2.32 system software.

### Chemicals and solvents

#### Pure samples

PAR, PSE and CET were kindly provided by GlaxoSmithKline (Cairo, Egypt). The purity of the samples was 99.40 ± 0.7780, 100.1 ± 0.4270 and 100.0 ± 0.2340, respectively, according to the reported method of analysis [10].

#### Market sample

Allercet Cold^®^ capsules were bought from a local pharmacy and were labeled to consist of 400 mg of PAR, 30 mg PSE and 10 mg CET per one capsule (Batch Number: B10518), manufactured by Global Napi pharmaceuticals (6th of October city, Egypt).

#### Solvents

Double distilled water.

### Standard solutions

Stock solutions with concentrations of 1000 µg mL^−1^ for PAR and CET and 4000 µg mL^−1^ for PSE using distilled water as a solvent were prepared. Next, fresh working solutions with concentrations of 100.0, 2000 and 100.0 µg mL^−1^ for PAR, PSE and CET, respectively, were made by diluting the corresponding stock solutions with distilled water.

### Procedures

#### Linearity

Accurately measured volumes of PAR (0.2500–2.500 mL), PSE (0.5000–6.000 mL) and CET (0.2000–4.500 mL) were accurately taken from their working standard solutions into series of volumetric flasks (10 mL), the volumes were completed with water to prepare final concentrations of 2.500–25.00 µg mL^−1^ for PAR, 100.0–1200 µg mL^−1^ for PSE and 2.000–45.00 µg mL^−1^ for CET. The prepared solutions were scanned from 200.0 to 400.0 nm and their absorption spectra were stored in the computer and were used in the manipulation steps of RDSM, DRZC and SDR.

##### Ratio subtraction–ratio difference method (RSDM)

**Fig. 2:**
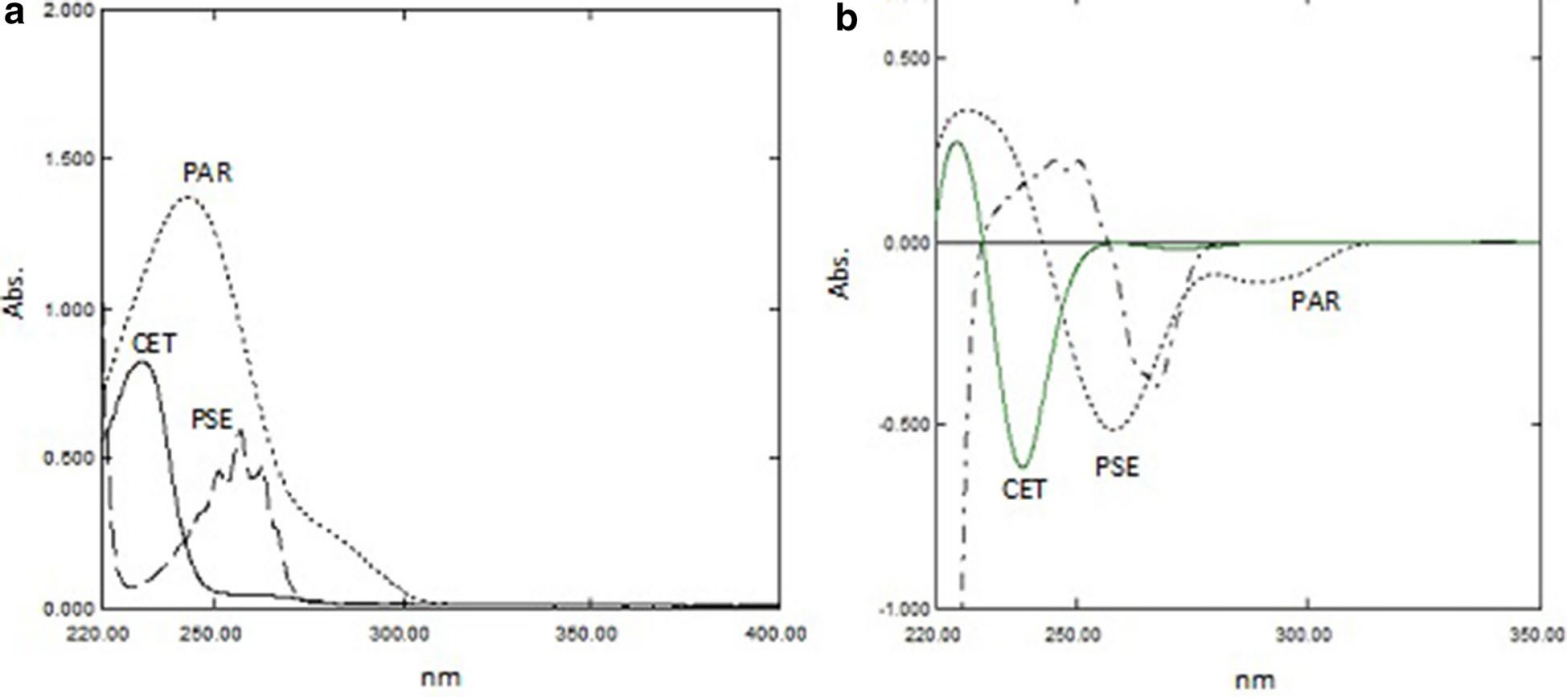
**a** Zero-order, **b** first derivative absorption spectra of 20.00, 600.0 and 20.0 µg mL^−1^ of PAR (^……^), PSE (- - - -) and CET (—), respectively

*For PAR* The first derivative (^1^D) spectrum of PAR is extended over the ^1^D spectra of PSE and CET, so it can be determined at wavelength 292.4 nm without the interference of the other two components as demonstrated in Fig. 2b. A calibration graph was constructed relating the absorbance of ^1^D of PAR at 292.4 nm against the corresponding concentrations and the regression equations were then computed.

*For PSE and CET* The stored zero order spectra (^0^D) of PSE were divided by the spectrum of 25.00 μg mL^−1^ CET, while (^0^D) spectra of CET were divided by the spectrum of 600.0 μg mL^−1^ of PSE. Calibration graphs for both PSE and CET were constructed by plotting the amplitude difference of the obtained ratio spectra between 257.0 and 230.0 nm for PSE and 228.0 and 257.0 nm for CET versus their corresponding concentrations and the regression equations were then computed.

##### Derivative ratio spectra–zero crossing spectrophotometric method (DRZC)

*For PAR* As under “Ratio subtraction–ratio difference method (RSDM)”.

*For PSE* The ^0^D spectra were divided by a standard spectrum of PAR (20.00 µg mL^−1^) and the ^1^D of the ratio spectra was obtained. PSE was determined from the ^1^D amplitudes at 252.0 nm which represented the zero-crossing point for CET. A calibration graph was constructed between the absorbance of ^1^D of PSE at 252.0 nm versus the corresponding concentrations and the regression equation was then computed.

*For CET* The spectra were divided by a standard spectrum of PAR (20.00 µg mL^−1^) and the third derivative (^3^D) of the ratio spectra was obtained. The concentration of CET was determined from ^3^D amplitudes at 237.0 nm which represented the zero-crossing point of PSE. A calibration graph was constructed between the absorbance of ^3^D of CET at 237.0 nm versus the corresponding concentrations and the regression equation was then computed.

##### Successive derivative ratio method (SDR)

*For PAR* The spectra were divided by the spectrum of 25.00 µg mL^−1^ CET. The ^1^D was computed for the ratio spectra and then a division process was carried out using the ^1^D spectrum of 600.0 µg mL^−1^ PSE/25.00 µg mL^−1^ CET as a divisor, and the second ratio spectra were obtained. Afterwards, the ^1^D was obtained allowing the concentration of PAR to be determined at the maximum amplitude at 310.2 nm. A calibration graph was created by plotting the amplitudes from the resulting curves at 310.2 nm against the corresponding concentrations and the regression equation parameters were then computed.

*For PSE* The spectra were divided by the spectrum of 25.00 µg mL^−1^ CET and ^1^D was computed for these ratio spectra. The obtained derivative of ratio spectra were then divided by ^1^D spectrum of 20.00 µg mL^−1^ PAR/25.00 µg mL^−1^ CET, where the second ratio spectra were obtained, and then the ^1^D was calculated. PSE was quantified at the minimum amplitude at 257.0 nm. A calibration graph was created by plotting the amplitudes from the resulting curves at 257.0 nm against the corresponding concentrations and the regression equation parameters were obtained.

*For CET* The spectra were divided by the spectrum of 600.0 µg mL^−1^ PSE and the ^1^D was computed for these ratio spectra. Next, the obtained derivative of ratio spectra were divided by ^1^D spectrum of 20.00 µg mL^−1^ PAR/600.0 µg mL^−1^ PSE, and the second ratio spectra were obtained. The ^1^D was calculated where the concentration of CET was determined at the minimum amplitude at 242.4 nm. A calibration graph was created by plotting the amplitudes from the resulting curves at 242.4 nm against the corresponding concentrations and the regression equation parameters were then computed.

##### Pure component contribution algorithm (PCCA)

Accurately measured volumes of PAR (0.2500–2.500 mL), PSE (0.5000–5.000 mL) and CET (0.5000–5.000 mL) were separately taken from their working standard solutions into a series of volumetric flasks (10 mL), the volumes were completed with water producing solutions with final concentration ranges of 2.500–25.00 µg mL^−1^ for PAR, 100.0–1000 µg mL^−1^ for PSE and 5.000–50.00 µg mL^−1^ for CET. The prepared solutions were scanned from 200.0 to 400.0 nm and the values of absorbance at *λ*_max_ were recorded. These absorbance values were used to create different plots for the three drugs against their corresponding concentrations and the regression equation parameters were then computed.

#### Analysis of laboratory-prepared mixtures

Different volumes of PAR, PSE and CET were accurately taken from their corresponding working standard solutions and placed in volumetric flasks of 10 mL capacity, finally, the volumes were completed using water. The prepared mixtures consisted of varying ratios of the three drugs. The laboratory prepared mixtures were scanned in the range from 200.0 to 400.0 nm and their absorption spectra were stored in the computer.

##### RSDM method

PAR was determined directly from the ^1^D at 292.4 nm (Δλ = 8.0, scaling factor 100), where PSE and CET have no contribution and concentrations of PAR were calculated from the obtained regression equation. The zero order absorption spectra of the laboratory prepared mixtures were divided by a carefully chosen concentration of PAR’ (20.00 µg mL^−1^) as a divisor. Thus, ratio spectra were produced represented by (PSE + CET)/PAR’ + constant, the values of these constants PAR/PAR’ in the plateau region (278.0–297.0 nm) were then subtracted, this is followed by multiplying the obtained ratio spectra by the divisor PAR’ (20.00 µg mL^−1^). Finally, the original spectra of PSE + CET were obtained for their determination by ratio difference.

In order to determine PSE and CET by ratio difference method, the same steps as under linearity “Ratio subtraction–ratio difference method (RSDM)” were performed and their concentrations obtained from the computed regression equations.

##### DRZC method

PAR was determined as under “RSDM method”. As for PSE and CET, the zero order absorption spectra of the laboratory prepared mixtures were divided by 20.00 µg mL^−1^ PAR. This was then followed by calculating the first and third derivatives for determining PSE and CET at 252.0 and 237.0 nm, respectively.

##### SDR method

Procedures for determining each drug in laboratory prepared mixture were applied as described under “Successive derivative ratio method (SDR)”.

##### PCCA method

*For PAR* The spectra of the mixtures were divided using the normalized spectrum of 45.00 µg mL^−1^ CET (αCET) as a divisor, then mean centering of the obtained ratio spectra was carried out and divided by MC (αPSE/αCET), the spectrum of 400.0 µg mL^−1^ of PSE was used. The produced curves were mean centered and divided by MC [MC (αPAR/αCET)/MC (αPSE/αCET)]. Constants representing the concentration of PAR in the mixtures were obtained and multiplied by the standard normalized spectrum of PAR and the absorbance at 245.0 nm were recorded in the obtained spectra.

*For PSE* The spectra mixtures were divided by the normalized spectrum of 45.00 µg mL^−1^ CET (αCET), and the obtained ratio spectra were then mean centered and divided by MC (αPAR/αCET), the spectrum of 10.00 µg mL^−1^ of PAR was used. Then, the produced curves were mean centered and divided by MC [MC (αPSE/αCET)/MC (αPAR/αCET)]. The obtained constants were multiplied by the standard normalized spectrum of PSE and the absorbance at 256.0 nm was recorded in the obtained spectra.

*For CET* The spectra of the mixtures were divided by the normalized spectrum of 10.00 µg mL^−1^ PAR (αPAR), the obtained ratio spectra were then mean centered and the produced curves were mean centered and divided by MC [MC (αCET/αPAR)/MC (αPSE/αPAR)]. The obtained constants were multiplied by the standard normalized spectrum of CET (αCET). The absorbance value was recorded at 230.0 nm in the obtained spectra.

Concentrations representing each drug was computed from their corresponding regression equation. The percentage recoveries, the mean percentage recovery and the standard deviations were calculated.

#### Application to pharmaceutical preparation

Ten Allercet Cold^®^ capsules were ground, mixed well and accurately weighed. An amount of the mixed powder equivalent to one capsule was accurately weighed and placed in a beaker; extracted with 3 × 30 mL water. The extract was sonicated for 15 min (for each extraction). Filtration was carried out into a 100-mL volumetric flask and completed to volume with the same solvent to obtain a solution (Stock 1) with the following concentrations 4000 µg mL^−1^ of PAR, 300.0 µg mL^−1^ of PSE and 100.0 µg mL^−1^ of CET. Then 1.000 mL from Stock 1 was accurately transferred into a 10-mL volumetric flask and diluted with water to prepare a solution (stock 2) with the concentration of 400.0 µg mL^−1^ of PAR, 30.00 µg mL^−1^ of PSE and 10.00 µg mL^−1^ of CET. An aliquot equivalent to 2.500 mL from Stock 2 was accurately transferred into a 100-mL volumetric flask. The solution was then spiked with 5.000 mL PSE and 2.000 mL CET from their corresponding working solutions and completed to volume with water forming a solution composed of 10.00, 100.8 and 2.250 µg mL^−1^ of PAR, PSE and CET, respectively. The procedure under “Analysis of laboratory-prepared mixtures” was carried out and the concentration of PAR, PSE and CET were computed from their corresponding regression equation.

The standard addition technique was performed by adding various amounts of pure standard drugs to the pharmaceutical dosage form before continuing the methods described previously.

## Results and discussion

Resolution of multicomponent mixtures which possess overlapping spectra is a challenging concern for analytical chemists. Although, chromatographic methods are usually chosen for the analysis of such mixtures, nevertheless, in the past few years the mathematical spectrophotometric methods have significantly substituted chromatography as they offer some advantages of being rapid, simple to apply, do not need any optimization of conditions, sensitive and cost-effective. Thus, we were encouraged to develop sensitive spectrophotometric techniques for the determination of PAR, PSE and CET simultaneously in their pure powders and dosage form with acceptable accuracy and precision especially as there are no reported spectrophotometric methods for their analysis.

The spectra of PAR, PSE and CET are severely overlapped as shown in Fig. 2a, therefore direct determination of the three drugs was not possible from measuring the absorption directly from zero order spectra. The proposed methods were successful in determining each component simultaneously without prior separation. They were also found to be simple, precise and reproducible.

### RSDM method

Ratio subtraction coupled with ratio difference (RSDM) is a successive spectrophotometric technique which was successful in the determination of the ternary mixture.

**Fig. 3:**
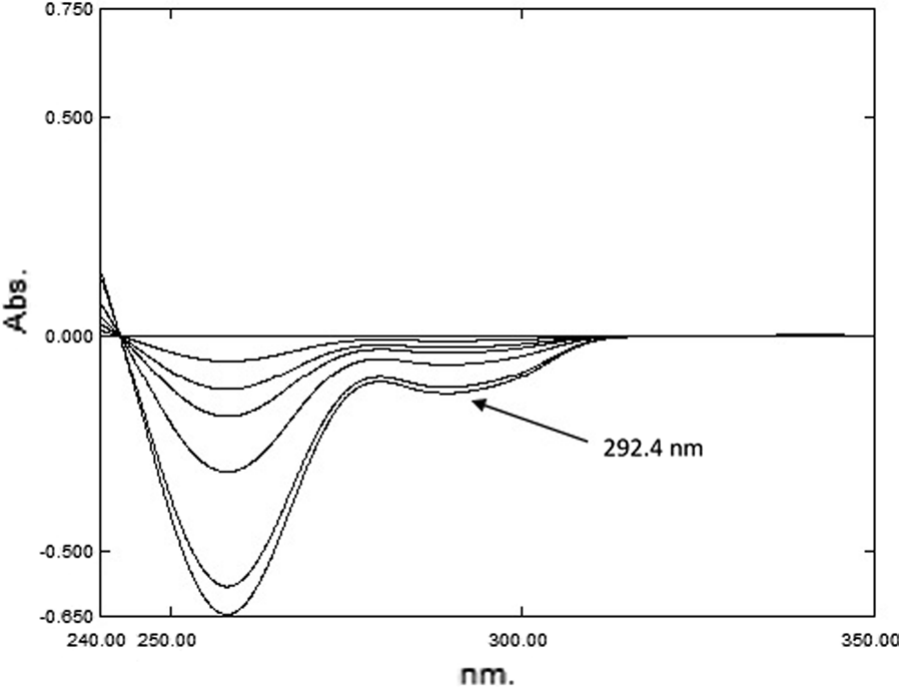
First order derivative spectra of Paracetamol

The ^1^D spectrum of PAR was extended over the ^1^D spectra of PSE and CET Fig. 2b, so PAR could be directly determined by utilizing the first derivative at 292.4 nm as the spectrum showed maximum absorbance value and no interfering signals from PSE and CET (∆λ = 8 and scaling factor = 10) as shown in Fig. 3 where its concentrations was determined from the computed regression equation. Then the spectrum of PAR was eliminated using RS [13] which could be applied as the spectrum of PAR was extended over the spectra of PSE and CET in their ternary mixture. To analyze PSE and CET in the mixtures, the zero order absorption spectra of the laboratory-prepared mixtures were divided by the spectrum of standard PAR (20.00 μg mL^−1^) as a divisor. The obtained ratio spectra represented PSE + CET/PAR + constant. The values of these constants in the plateau region (278.0–297.0 nm) were subtracted. The obtained spectra were then multiplied by spectrum of the divisor PAR (20.00 μg mL^−1^). Subsequently, the original spectra of PSE + CET were obtained which were used for their direct determination by utilizing RD.

**Fig. 4:**
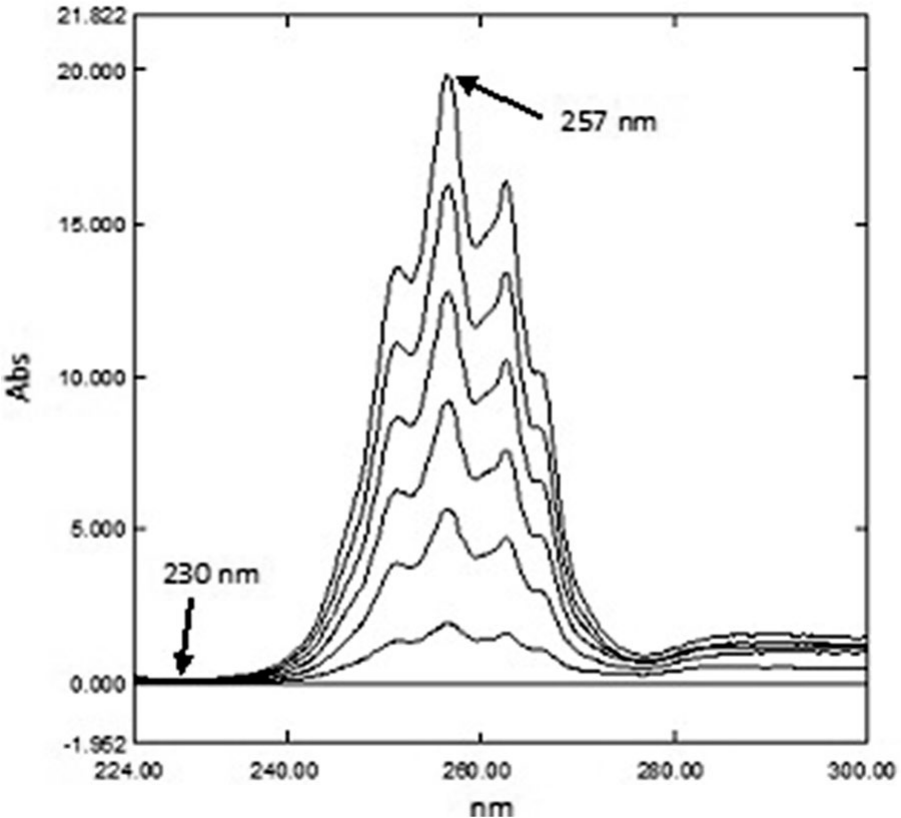
Ratio spectra of PSE using 25.00 µg mL^−1^ CET as divisor

**Fig. 5:**
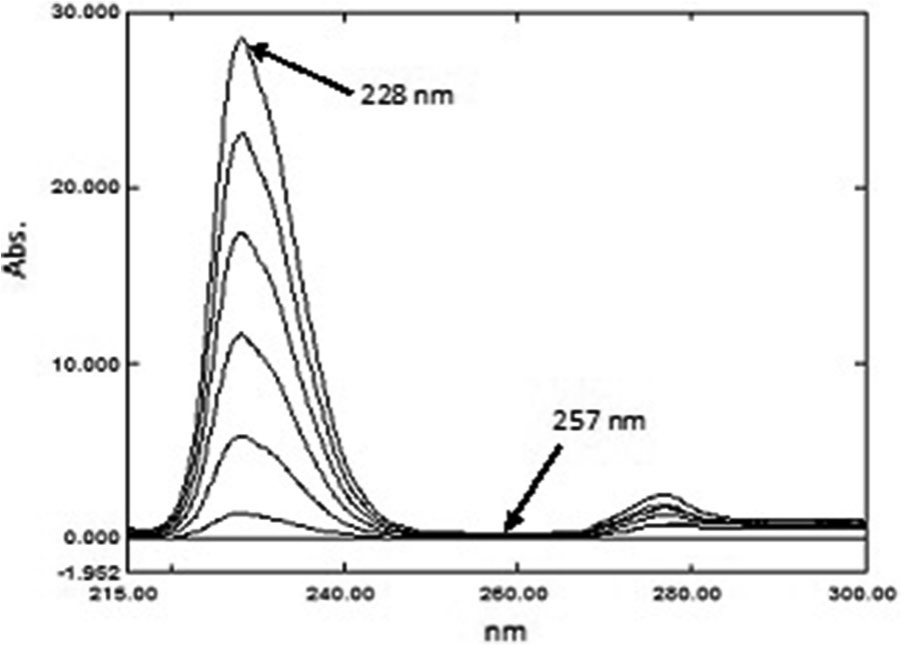
Ratio spectra of CET using 600.0 µg mL^−1^ PSE as divisor

To determine PSE and CET by the RD method [14] the zero order spectra of different laboratory prepared mixtures were divided by the absorption spectra of standard 600.0 μg mL^−1^ PSE and standard 25.00 μg mL^−1^ CET to obtain different ratio spectra as demonstrated in Figs. 4 and 5. Calibration curves were created by plotting the amplitude difference at 257.0 and 230.0 nm for PSE and the amplitude difference at 228.0 and 257.0 nm for CET versus their corresponding concentrations and the regression equations were calculated. The only requirement for the selection of these two wavelengths is the contribution of the two components at these two selected wavelengths where the ratio spectrum of the interfering component showed the same value (constant) whereas the component of interest shows a significant difference in these two ratio values at these two selected wavelengths [7].

### DRZC method

Nevado et al. [15], invented this method to resolve ternary mixtures. The method depends on the measurement of the amplitudes of the components of the mixture at the zero-crossing points in the derivative spectrum of the ratio spectra.

**Fig. 6:**
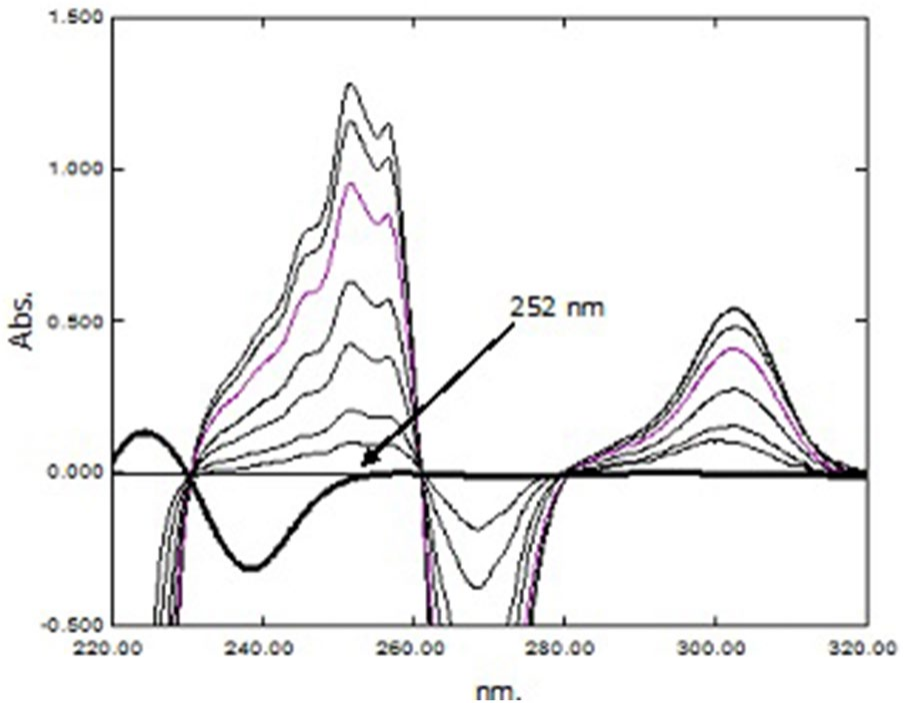
First derivative ratio spectra of PSE and CET using PAR (20.00 µg mL^−1^) as divisor

**Fig. 7:**
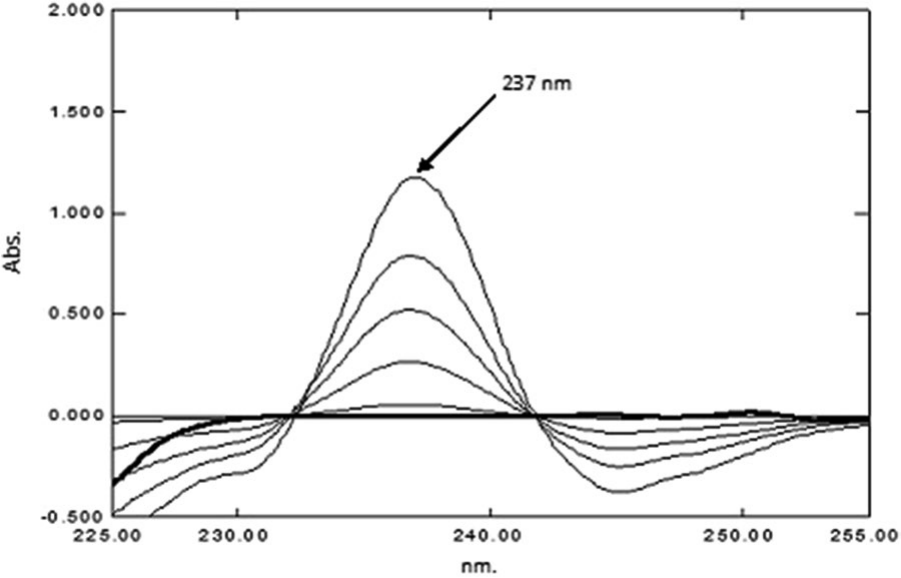
Third derivative ratio spectra of CET and PSE using PAR (20.00 µg mL^−1^) as divisor

PAR was determined as under “RSDM method”. Then, the spectra of the laboratory prepared mixtures were divided by the spectrum of standard PAR 20.00 µg mL^−1^ as a divisor to obtain the corresponding ratio spectra. Both the first derivative and third derivative of these ratio spectra were calculated. The concentration of PSE was proportional to the first order amplitudes at 252.0 nm (zero-crossing point for CET) as demonstrated in Fig. 6, while, the concentration of CET was proportional to the third order amplitudes at 237.0 nm (zero-crossing point of PSE) as shown in Fig. 7. The different concentrations of PSE and CET were determined from the computed regression equations.

### SDR method

Afkhami and Bahram [16] have proposed the SDR technique for the quantitation of ternary mixtures without prior separation. This method depends on successive steps; first the derivative of ratio spectra is calculated, and then these derivative ratio spectra are divided by the derivative ratio spectra of a divisor of the other two components. Finally, the derivative is computed for those obtained ratio spectra.

**Fig. 8:**
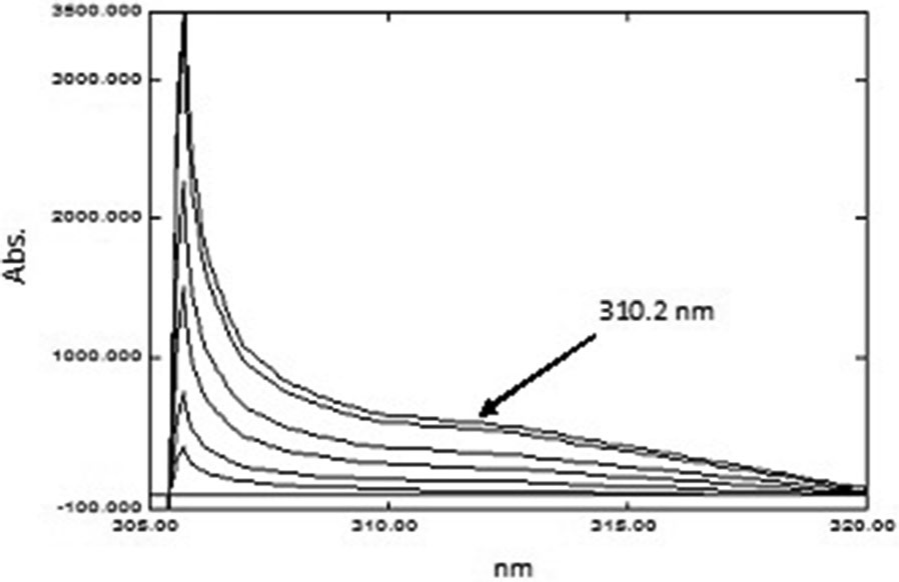
The vectors of the first derivative of the second ratio spectra for PAR in water

**Fig. 9:**
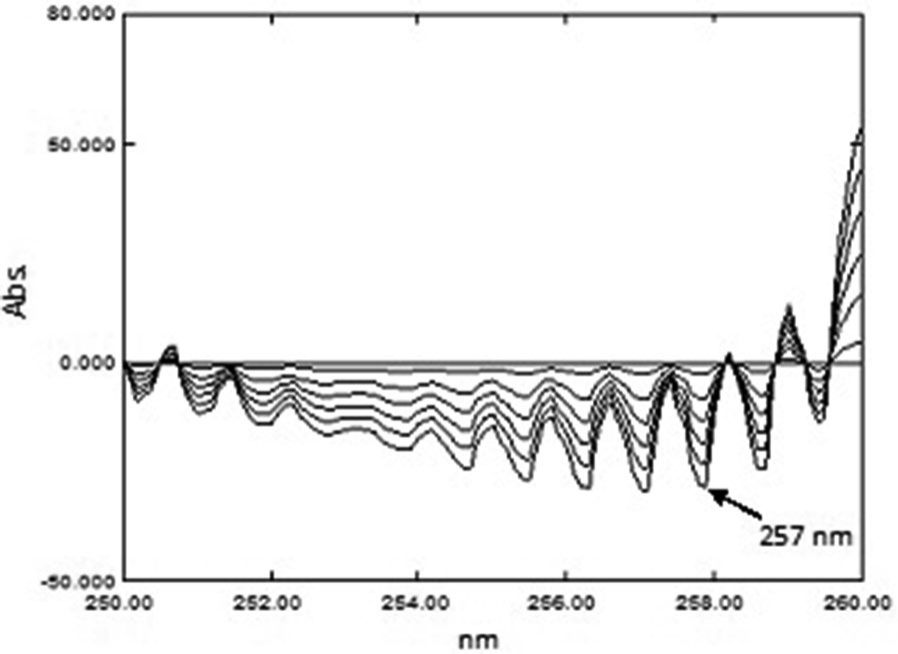
The vectors of the first derivative of the second ratio spectra for PSE in water

**Fig. 10:**
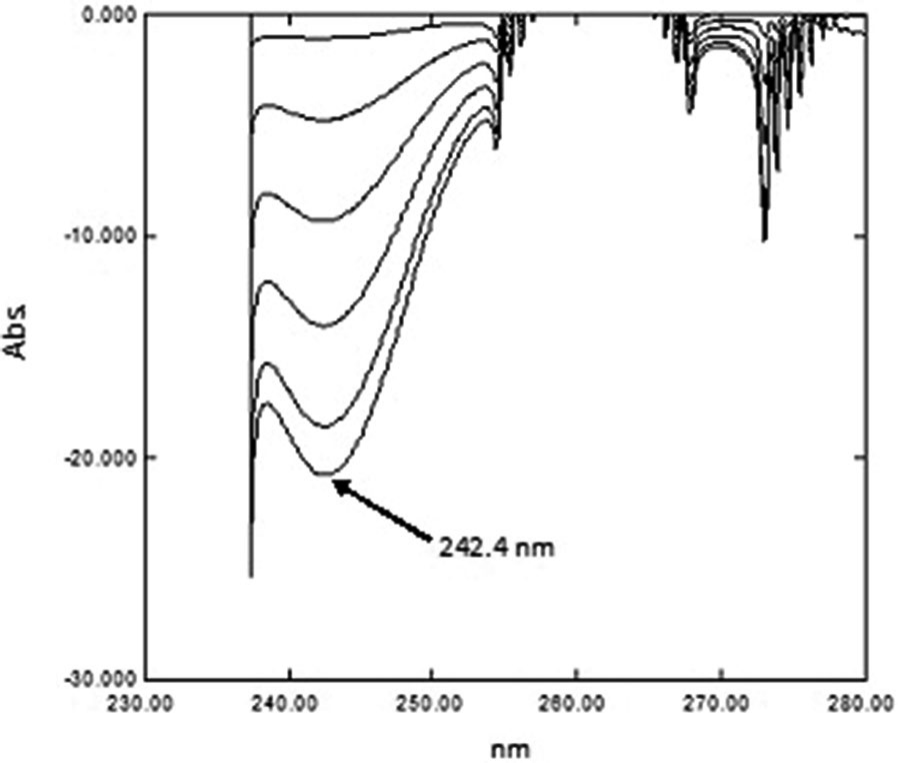
The vectors of the first derivative of the second ratio spectra for CET in water

For the determination of PAR and PSE; the absorption spectra of the laboratory prepared mixtures were divided by the spectrum of 25.00 μg mL^−1^ of CET and the first derivative was calculated for the ratio spectra (V1). For PAR, the vectors (V1) were divided by the ^1^D spectrum of 600.00 µg mL^−1^ PSE/25.00 µg mL^−1^ CET, thus the second ratio spectra were obtained (V2). Finally, the first derivative was calculated for these vectors (V2) where the concentration of PAR was determined at the maximum amplitude at 310.2 nm as illustrated in Fig. 8. For PSE, the vectors (V1) were divided by the D^1^ spectrum of 20.00 µg mL^−1^ PAR/25.00 µg mL^−1^ CET, where the second ratio spectra were obtained (V3). First derivative was calculated for these vectors (V3) and the concentration of PSE was determined by measuring the maximum amplitude at 257.0 nm as demonstrated in Fig. 9. To determine CET, the absorption spectra of the laboratory prepared mixtures were divided by the spectrum of 600.0 μg mL^−1^ PSE followed by calculating the first derivative for these ratio spectra. The obtained derivative of ratio spectra were then divided by ^1^D spectrum of 20.00 µg mL^−1^ PAR/600.0 µg mL^−1^ PSE, thus, the second ratio spectra were obtained. Finally, the concentration of CET was determined by measuring the maximum amplitude at 242.4 nm as shown in Fig. 10. According to Afkhami and Bahram [16], there are no limitations regarding the selection of wavelengths for the construction of the calibration graphs therefore the wavelengths used were selected after trying several others and the selected ones demonstrated the best regression parameters.

For all the proposed methods; the chosen divisor to settle between the lowest noise level and highest sensitivity and obtain optimal findings regarding average recovery percent for the analysis of laboratory prepared mixtures were analyzed. To refine D^1^ method, many smoothing and scaling factors were tried, where a smoothing *Δλ *= 8 and a scaling factor = 10 demonstrated acceptable signal to noise ratio and good resolution of spectra.

### PCCA method

**Fig. 11:**
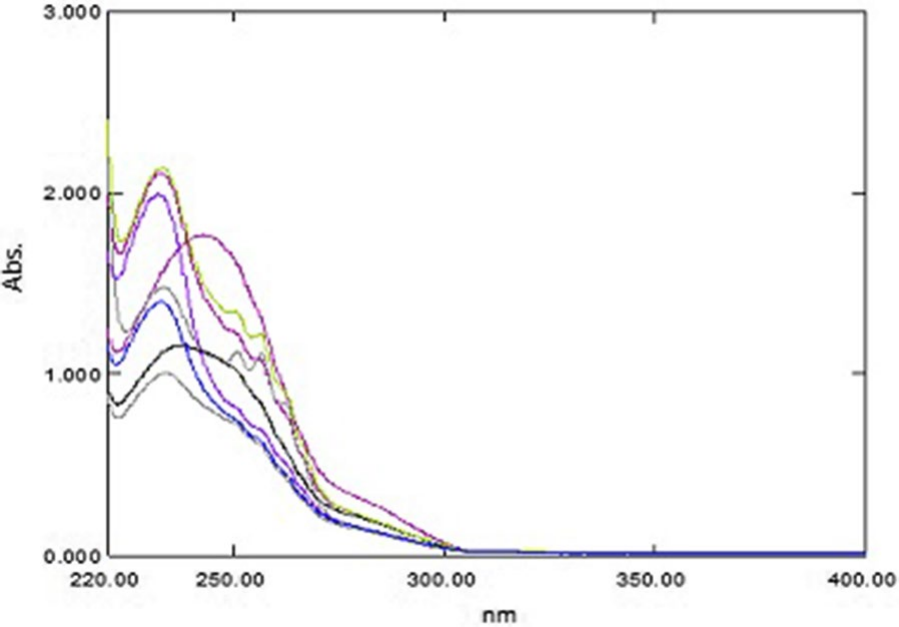
The spectra of laboratory prepared mixtures of paracetamol, pseudoephedrine hydrochloride and cetirizine dihydrochloride

**Fig. 12:**
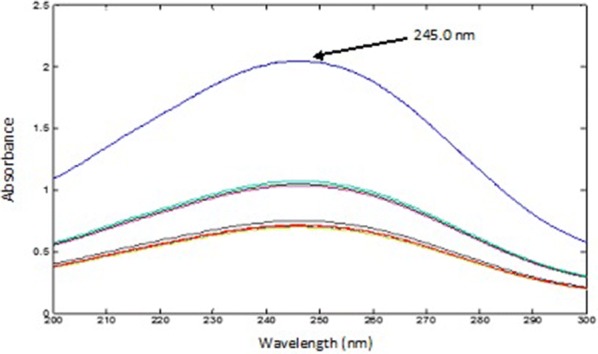
The pure contribution of paracetamol in the prepared mixtures

The UV absorption spectra of PAR, PSE and CET, Fig. 2a showed sever overlapping as a result the determination of the proposed drugs using conventional spectrophotometric methods was not possible. An algorithm able to resolve and extract the pure component contribution from their mixture signal without any special requirements was applied. The PCCA method is characterized by its varying applications, as it has no limitations, as opposed to other methods which require the extention of one spectrum over the others or the presence of zero-crossing or isoabsorptive points. The method is based on obtaining the pure component from its mixture and its determination at its λ_max_ providing maximum sensitivity, accuracy and precision results. For quantifying PAR in lab prepared ternary mixtures and dosage forms; the spectra of the mixtures, Fig. 11 were divided by the normalized spectrum of CET (αCET), the obtained ratio spectra were then mean centered and divided by MC (αPSE/αCET). Mean centering was applied on the produced curves then divided by MC [MC (αPAR/αCET)/MC (αPSE/αCET)]. Constants which represent the concentration of PAR in the mixtures were obtained. At the final step, the constants were multiplied by the standard normalized spectrum of PAR (αPAR) and the pure contribution of PAR in each mixture was obtained, Fig. 12. The estimated absorbance value of each of the obtained spectra at 245.0 nm was used for determining the concentration of PAR from the regression equation of PAR standard solutions.

**Fig. 13:**
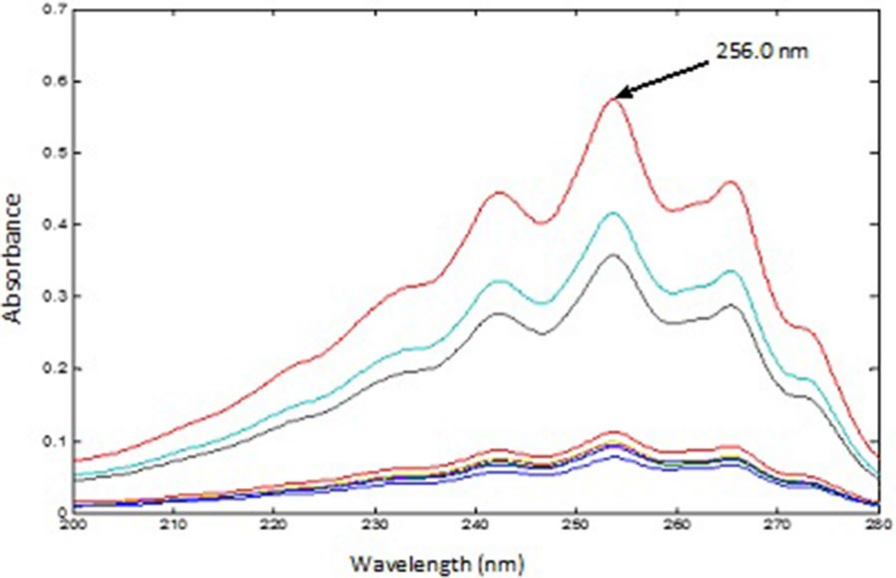
The pure contribution of pseudoephedrine hydrochloride in the prepared mixtures

Following the procedure previously stated, PSE was determined in synthetic mixtures and dosage forms; the spectra of the mixtures were divided by the normalized spectrum of CET (αCET), and the obtained ratio spectra were then mean centered and divided by MC (αPAR/αCET). Then, the produced curves were mean centered and divided by MC [MC (αPSE/αCET)/MC (αPAR/αCET)]. Constants which represent the concentration of PSE in the mixtures were obtained. Lastly, the resulting constants were multiplied by the standard spectrum of PSE (αPSE) and the pure contribution of PSE in each mixture was obtained, Fig. 13. The estimated absorbance value of each of the obtained spectra at 256.0 nm was used for calculating the concentration of PSE from the previously calculated regression equation of PSE.

**Fig. 14:**
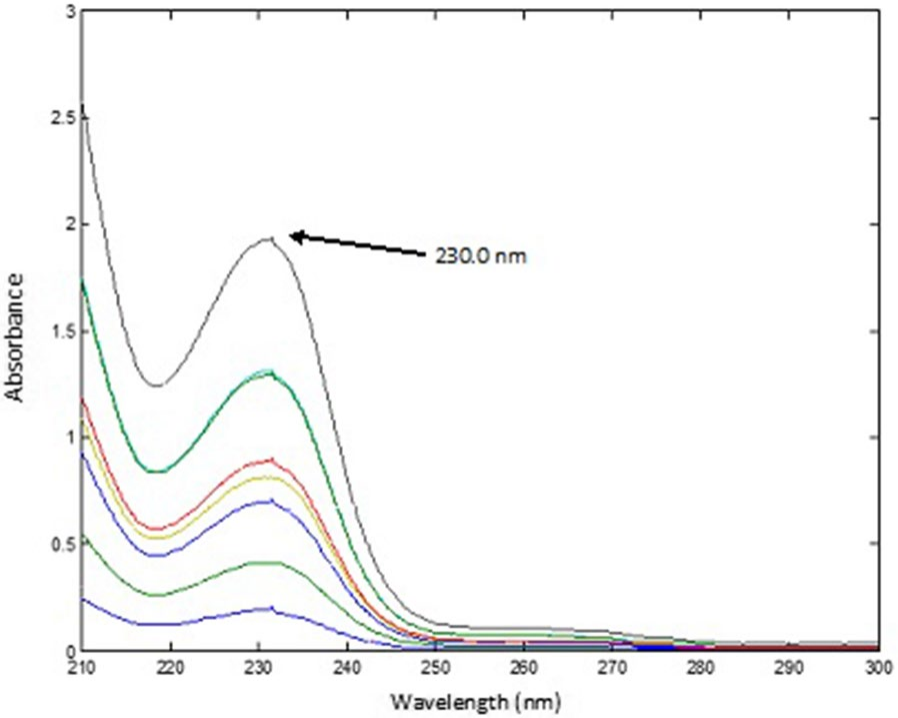
The pure contribution of cetirizine dihydrochloride in the prepared mixtures

Finally, for the determination of the concentration of CET in synthetic mixtures and dosage form samples; the spectra of the mixtures were divided by the normalized spectrum of PAR (αPAR), the obtained ratio spectra were then mean centered and divided by MC (αPSE/αPAR). Then, the produced curves were mean centered and divided by MC [MC (αCET/αPAR)/MC (αPSE/αPAR)]. Constants which represent the concentration of CET in the mixtures were obtained. The obtained constants were multiplied by the standard spectrum of CET (αCET) and the pure contribution of CET in each mixture was obtained, Fig. 14. The estimated absorbance value of each of the obtained spectra at 230.0 nm was used for calculating the concentration of CET from the previously calculated regression equation of CET standard solutions.

The contribution of each of PAR, PSE and CET were resolved and their contribution in each mixture was extracted, from which the absorbance values of the components were determined at their λ_max_ which are associated with maximum sensitivity, highest accuracy and precision and lowest error.

## Method validation

Validation according to ICH guidelines were applied for the suggested methods [18] where good results were obtained.

### Range and linearity

The calibration curves of the different proposed methods were handled on three different days in order to evaluate the linearity. The analytical data of the calibration graph were demonstrated in Table 1.

**Table 1:** Regression and validation parameters of the proposed spectrophotometric methods for the determination of PAR, PSE and CET

Parameters	RSDM method			SDR method			DRZC method			PCCA method		
	PAR	PSE	CET	PAR	PSE	CET	PAR	PSE	CET	PAR	PSE	CET
Linearity range (µg mL^−1^)	2.500–25.00	100.0–1100	2.000–50.00	2.500–25.00	100.0–1200	2.000–45.00	2.500–25.00	100.0–1200	2.000–45.00	2.500 –25.00	100.0 –900.0	5.000–50.00
Slope	5.500 × 10^−3^	1.740 × 10^−2^	5.620 × 10^−1^	2.930 × 10^−1^	2.860 × 10^−2^	4.600 × 10^−1^	5.500 × 10^−3^	1.100 × 10^−3^	2.610 × 10^−2^	6.160 × 10^−2^	9.000 × 10^−4^	3.140 × 10^−2^
Intercept	1.100 × 10^−3^	2.130 × 10^−1^	2.560 × 10^−1^	2.930 × 10^−1^	1.630 × 10^−2^	1.560 × 10^−1^	7.000 × 10^−4^	2.200 × 10^−3^	9.000 × 10^−4^	6.000 × 10^−3^	6.100 × 10^−3^	7.900 × 10^−3^
SE of slope	1.210 × 10^−5^	8.140 × 10^−5^	3.060 × 10^−3^	1.100 × 10^−1^	1.680 × 10^−4^	1.650 × 10^−3^	3.030 × 10^−5^	7.150 × 10^−6^	8.660 × 10^−5^	6.850 × 10^−4^	4.270 × 10^−6^	5.310 × 10^−5^
SE of intercept	2.040 × 10^−4^	5.620 × 10^−2^	9.270 × 10^−2^	1.740	1.300 × 10^−1^	4.770 × 10^−2^	4.980 × 10^−4^	5.430 × 10^−3^	2.730 × 10^−3^	1.040 × 10^−2^	2.69 × 10^−3^	1.70 × 10^−3^
Correlation coefficient (r)	1.000	1.000	1.000	1.0000	1.000	1.0000	1.000	1.000	1.000	1.000	1.000	1.000
Accuracy (n = 6) mean ± SD	100.4 ± 0.8000	99.40 ± 0.3900	100.1 ± 0.8700	99.57 ± 1.090	99.88 ± 1.280	100.2 ± 0.7100	100.9 ± 0.7100	99.68 ± 1.090	100.1 ± 0.6300	100.7 ± 1.529	98.82 ± 0.5310	100.4 ± 0.3980
Precision (n = 3 × 3) (RSD %)^a^ Repeatability intermediate precision	0.07800 1.220	0.05280 1.010	0.07700 1.230	0.5280 0.9050	0.09100 0.8370	0.1550 1.300	0.07800 1.220	0.2250 0.4290	0.2200 0.5200	0.1560 0.8810	0.2260 0.9970	0.1140 0.8000
LOD^b^	0.1520	12.91	0.6570	0.2850	17.42	0.4470	0.3760	23.13	0.3690	0.6430	9.900	0.2210
LOQ^b^	0.4610	39.11	1.990	0.8640	52.78	1.350	1.140	70.08	1.120	1.948	30.00	0.6690

^a^Relative standard deviations (RSD) of three concentrations, the concentrations were as follows: PAR (5.000, 10.00, 25.00 µg mL^−1^), PSE (100.0, 600.0, 1000 µg mL^−1^) and CET (5.000, 15.00, 35.00 µg mL^−1^)

^b^LOD = 3.3 × Standard deviation of residuals/slope; LOQ = 10 × Standard deviation of residuals/slope

### Limits of detection (LOD) and quantification (LOQ)

The LOD and LOQ were calculated (Table 1) for the studied drugs using the proposed techniques according to the following equations:

$${\text{LOD }} = { 3}. 3 { }*{\text{ SD of residuals}}/{\text{Slope}}$$

$${\text{LOQ }} = { 1}0 \, *{\text{ SD of residuals}}/{\text{Slope}}$$

### Accuracy

The proposed methods were utilized for the analysis of different solutions of PAR, PSE and CET in order to validate the accuracy. The concentrations were deduced from the corresponding regression equations, then the percentage recoveries and standard deviation were calculated. The results demonstrated in Table 1 have assured the accuracy of all methods.

### Repeatability and intermediate precision

Three concentrations of PAR (5.000, 10.00, 25.00 µg mL^−1^), PSE (100.0, 600.0, 1000 µg mL^−1^) and CET (5.000, 15.00, 35.00 µg mL^−1^) were analyzed three times intra-daily and inter-daily (on three different days) using the proposed spectrophotometric methods. The relative standard deviations were calculated proving the precision of the methods (Table 1).

### Selectivity

The methods’ selectivity was accomplished by analyzing different laboratory prepared mixtures with varying concentrations of the three drugs within the linearity range. Acceptable results were illustrated in Table 2.

**Table 2:** Analysis of laboratory prepared mixtures by the proposed spectrophotometric methods

	RSDM method	DRZC method	SDR method	PCCA method
PAR^a^ (Mean ± SD)	100.7 ± 1.890	101.9 ± 1.060	101.8 ± 0.8600	100.4 ± 1.390
PSE^a^ (Mean ± SD)	99.69 ± 0.8400	99.59 ± 1.010	99.04 ± 1.200	98.76 ± 0.6800
CET^a^ (Mean ± SD)	99.38 ± 1.550	100.2 ± 0.6300	98.95 ± 1.300	100.4 ± 1.980

^a^Average of 6 experiments

### Application of the proposed methods in Allercet^®^ capsules

The suggested procedures were used for the determination of PAR, PSE and CET in Allercet cold^®^ capsules. The obtained recovery and standard deviation have established the absence of interference from the excipients. Standard addition technique was also applied to further assure the validity of the proposed methods as demonstrated in Table 3.

**Table 3:** Application of standard addition technique to the analysis of PAR, PSE and CET in Allercet Cold^®^ capsules using the proposed spectrophotometric methods

Drug	RSDM method			SDR method			DRZC method			PCCA method		
	Claimed amount taken	Added	Recovery %^a, b^	Claimed amount taken	Added	Recovery %^a, b^	Claimed amount taken	Added	Recovery %^a, b^	Claimed amount taken	Added	Recovery %^a, b^
PAR	10.00 (µg mL^−1^)	5.000	104.6	10.00 (µg mL^−1^)	5.000	105.9	10.00 (µg mL^−1^)	5.000	101.1	10.00 (µg mL^−1^)	5.000	103.1
		10.00	104.0		10.00	104.9		10.00	100.8		10.00	103.8
		15.00	105.1		15.00	105.3		15.00	101.2		15.00	102.3
	Mean ± SD		104.6 ± 0.5210	Mean ± SD		105.4 ± 0.5490	Mean ± SD		101.0 ± 0.2330	Mean ± SD		103.1 ± 0.7360
PSE	100.75 (µg mL^−1^)*	50.00	104.0	100.8 (µg mL^−1^)	50.00	105.6	100.8 (µg mL^−1^)	50.00	104.1	100.8 (µg mL^−1^)	50.00	102.9
		100.0	103.2		100.0	104.3		100.0	105.0		100.0	102.8
		200.0	103.2		200.0	105.0		200.0	104.9		200.0	102.4
	Mean ± SD		103.5 ± 0.4780	Mean ± SD		105.0 ± 0.6500	Mean ± SD		104.6 ± 0.5030	Mean ± SD		102.7 ± 0.2310
CET	2.250 (µg mL^−1^)*	2.000	105.7	2.250 (µg mL^−1^)	2.000	105.9	2.250 (µg mL^−1^)	2.000	103.8	2.250 (µg mL^−1^)	2.000	101.9
		2.500	104.5		2.500	104.5		2.500	102.9		2.500	101.0
		10.00	105.6		10.00	104.0		10.00	102.8		10.00	101.1
	Mean ± SD		105.3 ± 0.6970	Mean ± SD		104.8 ± 1.018	Mean ± SD		103.2 ± 0.5460	Mean ± SD		101.3 ± 0.4800

* Amount spiked was 100.00 µg mL^−1^ for PSE and 2.00 µg mL^−1^ for CET

^a^Average of three experiments

^b^ Recovery of the claimed amount taken

## Statistical analysis

The results of the analysis of the pure drugs obtained from the proposed methods were compared to those obtained by applying the reference method [10] where no significant difference was observed from the calculated t- and F values, Table 4.

**Table 4:** Statistical comparison of the results obtained by the proposed spectrophotometric methods and reference method for the determination of PAR, PSE and CET

Parameter	PAR					PSE					CET				
	RSDM	SDR	DRZC	PCCA	Reference method	RSDM	SDR	DRZC	PCCA	Reference method	RSDM	SDR	DRZC	PCCA	Reference method
Mean	99.72	100.1	99.80	100.7	99.40	100.3	100.2	99.39	99.92	100.1	100.1	99.82	99.97	100.1	100.0
SD	0.5870	0.6200	0.9230	1.384	0.7780	0.8050	0.7310	1.130	1.110	0.4270	0.5720	0.5290	0.4290	0.5390	0.2340
N	6	6	6	6	4	6	6	7	6	4	6	6	6	6	4
Variance	0.3450	0.3840	0.8520	1.915	0.6050	0.6480	0.5340	1.277	1.232	0.1820	0.3270	0.2800	0.1840	0.2910	0.05500
Student’s t	0.7570 (2.310)	1.540 (2.310)	0.7130 (2.310)	1.660 (2.310)		0.3310 (2.310)	0.9290 (2.310)	1.200 (2.260)	2.070 (2.310)		0.2380 (2.310)	0.6830 (2.310)	0.2100 (2.310)	0.1290 (2.310)	
F	1.760 (5.410)	1.580 (5.410)	1.410 (9.010)	3.170 (9.010)		3.560 (9.010)	2.930 (9.010)	7.020 (8.940)	6.770 (9.010)		5.950 (9.010)	5.090 (9.010)	3.350 (9.010)	5.280 (9.010)	

Figures between parentheses represent the corresponding tabulated values of t and F at P = 0.05

The reported method is an HPLC method using C18 column, a mobile phase composed of 25 mM phosphate buffer (pH = 5): methanol: acetonitrile (30:60:10, *v/v/v*)

## Conclusion

The introduced study has demonstrated the application of simple and accurate mathematical based spectrophotometric methods for the analysis of the ternary mixture; paracetamol, pseudoephedrine and cetirizine in bulk and in Allercet Cold^®^ capsules the available dosage form in the Egyptian market. These methods have neither required any chemical pretreatment for the analyte nor demanded the availability of a complicated or advanced instrument. Moreover, these methods have employed the use of water as a solvent, thus, they could be considered as eco-friendly methods of analysis. The privileges of each method as well as the essential conditions for applying each method were discussed. All the developed methods were completely validated in accordance to the ICH guidelines proving their accuracy and precision. Furthermore, the selectivity of the methods was proved through the analysis of both laboratory prepared mixtures of the analytes as well as the dosage form were the commonly used excipients or additives have not interfered in the analysis as demonstrated from the consistency of the obtained results. Finally, the simplicity and accuracy of the developed methods could allow their effective utilization in the routine analysis of the investigated analytes in quality control laboratories.

## Abbreviations

CET: cetirizine dihydrochloride
DRZC: derivative ratio–zero crossing method
HPLC–UV: high performance liquid chromatography–ultra violet detection
PAR: paracetamol
PCCA: pure component contribution algorithm
PSE: pseudoephedrine hydrochloride
RSDM: ratio subtraction–ratio difference method
SDR: successive derivative ratio
